# A University Diploma for Nurses

**Published:** 1921-06-25

**Authors:** 


### A UNIVERSITY DIPLOMA FOR NURSES.

What must be regarded as a notable advance in the status of the nursing profession is the decision of the University of Leeds to grant a Diploma in Nursing. A preliminary draft of the Regulations is published elsewhere in this issue, but it may be stated that they follow very closely the tutorial scheme which has been in operation for some years at the Leeds General Infirmary. This has recently been perfected and raised to a high standard, and the instruction has been given mostly by members of the University staff. It has aimed at giving elementary training on sound lines; that is to say, efforts have been directed to ensure that every nurse shall have a really efficient education on the theoretical side of her profession as well as on the practical side. Every lecture-course is followed by a class examination, which a nurse is required to pass before proceeding to a general final examination, which she is also required to pass before receiving her Nursing Certificate.

It was felt by those responsible for training in the Leeds School that the time had come when the University might be asked to give official recognition to the fact that the Profession of Nursing is something more than a mere craft-training, and that it possesses an important academic side. This has now been done by granting a Diploma after examination, and it is a matter for congratulation to the Leeds School that the regulations adopted for the Diploma are essentially those necessary for qualifying for the Leeds Infirmary certificate. In addition to this, candidates will be required to take some part of their academic training in the University, and this must include a course of study in some special subject, social economics being the one more particularly indicated. Much of the credit for this step, so long and so ardently desired, is due to the untiring work of Professor J. Kay Jamieson, Dean of the Faculty of Medicine, Leeds University, and to Dr. Maxwell Telling, Honorary Physician to the Leeds General Infirmary.

It is contemplated that the Diploma will be in the nature of an Honours Certificate, of which the present general training certificate may be taken to represent the pass standard. Consequently no candidate can sit for the Diploma who has not completed her nursing training and examination. It is to be particularly noted that the Diploma regulations insist that this training shall be of four years' duration instead of the three years' which still generally obtains. The Leeds Infirmary was one of the first nursing schools in the country to insist upon the four years' standard, and anyone in touch with the actualities of modern nursing training, both on the practical and academic side, knows that the longer period is the more satisfactory. The regulations have been framed to enable nurses from schools other than Leeds to take the Diploma, though it will naturally be specially convenient, geographically and otherwise, for Leeds trained nurses. This is in the true university spirit, and it is to be hoped that many nurses will avail themselves of this opportunity.

It may be predicted with certainty that many important results will follow from this notable advance. In the first place, other universities will doubtless follow the example of Leeds. It will serve to develop a body of highly-trained nurses who are specialising in the academic and teaching side of their profession for whom there is a great and growing need, and to give all those seeking high administrative posts a guarantee of educational and scientific attainment, as well as sound practical work. But the chief gain of all is that it will testify to the general world that nursing is really a learned profession, and this testimony will tend to attract to it girls of really good education and scientific ambitions now that the university path lies open. There is no doubt that up to now the educational standard of nurses has been too low and the tendency has been, inevitably, for many girls of greater capacity to ignore the profession as a sufficiently satisfactory outlet for their energies and intellectual capacity. One hopes to see this changed, and now that the age of entering upon training, so long kept unnecessarily high, has been reduced to twenty years (and in some schools to nineteen), there is no reason why girls of high ability and general culture should not pass at once from their public-school to the hard but satisfying training of a nursing career. The road now lies open, and if the profession makes use of the opportunity thus presented there is no doubt that the provision of Degrees in nursing will soon be an accomplished fact.

